# Proteomic Analysis of the Meniscus Cartilage in Osteoarthritis

**DOI:** 10.3390/ijms22158181

**Published:** 2021-07-30

**Authors:** Jisook Park, Hyun-Seung Lee, Eun-Bi Go, Ju Yeon Lee, Jin Young Kim, Soo-Youn Lee, Dae-Hee Lee

**Affiliations:** 1 Samsung Biomedical Research Institute, Samsung Medical Center, Sungkyunkwan University School of Medicine, Seoul 06351, Korea; js944837@hanmail.net (J.P.); geb926@naver.com (E.-B.G.); 2 Department of Laboratory Medicine and Genetics, Samsung Medical Center, Sungkyunkwan University School of Medicine, Seoul 06351, Korea; hyunseung1011.lee@samsung.com; 3 Research Center for Bioconvergence Analysis, Korea Basic Science Institute, Cheongju 28119, Korea; jylee@kbsi.re.kr (J.Y.L.); jinyoung@kbsi.re.kr (J.Y.K.); 4 Department of Clinical Pharmacology and Therapeutics, Samsung Medical Center, Seoul 06351, Korea; 5 Department of Health Science and Technology, Samsung Advanced Institute of Health Science and Technology, Sungkyunkwan University, Seoul 06351, Korea; 6 Department of Orthopedic Surgery, Samsung Medical Center, Sungkyunkwan University School of Medicine, Seoul 06351, Korea

**Keywords:** meniscus, proteomics, MRM, ECM

## Abstract

The distribution of differential extracellular matrix (ECM) in the lateral and medial menisci can contribute to knee instability, and changes in the meniscus tissue can lead to joint disease. Thus, deep proteomic identification of the lateral and medial meniscus cartilage is expected to provide important information for treatment and diagnosis of various knee joint diseases. We investigated the proteomic profiles of 12 lateral/medial meniscus pairs obtained from excess tissue of osteoarthritis patients who underwent knee arthroscopy surgery using mass spectrometry-based techniques and measured 75 ECM protein levels in the lesions using a multiple reaction monitoring (MRM) assay we developed. A total of 906 meniscus proteins with a 1% false discovery rate (FDR) was identified through a tandem mass tag (TMT) analysis showing that the lateral and medial menisci had similar protein expression profiles. A total of 131 ECM-related proteins was included in meniscus tissues such as collagen, fibronectin, and laminin. Our data showed that 14 ECM protein levels were differentially expressed in lateral and medial lesions (*p* < 0.05). We present the proteomic characterization of meniscal tissue with mass spectrometry-based comparative proteomic analysis and developed an MRM-based assay of ECM proteins correlated with tissue regeneration. The mass spectrometry dataset has been deposited to the MassIVE repository with the dataset identifier MSV000087753.

## 1. Introduction

The knee is the largest hinge joint associated with weight-bearing and movement in the human body. The meniscus is at type of fibrocartilage, has properties similar to those of bone, and serves many important biomechanical functions such as stabilizing joints and absorbing damage. The knee contains medial and lateral menisci; the medial meniscus tends to experience more frequent damage than the lateral meniscus due to its anatomical structure and articular mechanism [1]. Such damage includes a tear, which occurs when placing excessive pressure on or twisting the knee joint. Meniscal degeneration and surgically removed meniscus are risk factors for osteoarthritis [2], while aging of the meniscus results in molecular and cellular changes. Thus, an understanding of the proteomic changes in the medial and lateral meniscal tissues will contribute to uncovering the cause of knee-related degenerative disease.

The extracellular matrix (ECM) plays an essential role in many processes, including cell-cell adhesion, signaling, and tissue repair. The meniscal ECM consists of water, fibrillar protein, proteoglycans, and adhesive glycoproteins, the activities of which are not fully understood.

To date, meniscus proteomics studies are rare, although information on hyaline cartilage protein has been reported [3,4,5]. Recently, global proteomic investigations have been performed on medial meniscal tissues to analyze the global protein expression profiles in radial zones using mass spectrometric technologies. Pairwise comparisons of the medial/lateral meniscus is a potentially unique model for the study of cartilage proteomics in osteoarthritis genesis and progression, because the anatomical structure and biomechanical configuration of the knee is typically associated with much more severe cartilage loss on the predominantly weight-bearing medial compartment. Also, pairwise comparisons of the lateral/medial meniscus have advantages independent of heterogeneous factors such as age, sex, osteoarthritis severity, underlying disease, and individual variability.

Deep proteomics might be a powerful tool in characterization of meniscal tissues [6,7]. Therefore, we investigated the proteome profile of meniscal tissue of lateral and medial lesions and characterized the extracellular matrix proteins. Herein, we provide extracellular matrix data and use multiple reaction monitoring (ECM-MRM) assays to compare expression levels in two lesion types.

## 2. Results

### 2.1. Proteomic Analysis of Meniscal Tissues

Semi-quantitative analysis was performed on meniscal tissues to compare the protein compositions of lateral and medial lesions using six plex-tandem mass tag (TMT) labeling. A total of 7876 unique peptides representing 906 proteins (903 genes) were identified in the meniscal tissues. Here, we found that the lateral and medial menisci have similar protein expression profiles. Most proteins showed similar concentrations in the lateral and medial lesions. Cartilage oligomeric matrix protein (COMP), cartilage intermediate layer protein (CILP), aggrecan (CAN), and 7 types of collagens (collagen type I, III, VI, XII, XIV, XV, and XVIII) were dominantly identified in both groups (Supplementary Table S1). A total of 24 proteins showed more than a two-fold concentration difference between the two lesions on TMT analysis. Of them, eleven proteins were ECM proteins (46% of the total), and further verified using MRM assay (Supplementary Table S2).

### 2.2. Characterization of ECM Proteins in the Meniscus

Among 906 proteins identified, 131 ECM or ECM-associated proteins were identified in meniscal tissues by MatrisomeDB 2.0 (http://www.pepchem.org/matrisomedb accessed on 31 May 2021) that integrated experimental proteomic data on the ECM composition of meniscal tissues (Supplementary Figure S1A). These proteins were categorized into 41 ECM glycoproteins, 14 proteoglycans, 11 collagen isoforms, 34 ECM regulators, 16 ECM affiliated proteins, and 15 secreted factors (Supplementary Figure S1B). To identify their biological function, we conducted functional enrichment analysis of the 131 proteins (130 genes) using Funrich software (Supplementary Figure S1C). Cell growth and/or maintenance (37.7% of genes) and protein metabolism (23.1%) were enriched according to the biological process. Extracellular matrix (82%) and ECM-related (38.3%) genes were dominantly enriched in the cellular component. According to molecular function, 34.6% of genes were categorized in the extracellular matrix structural constituent. These genes were highly involved in biological pathways such as the epithelial to mesenchymal transition (34%) and beta3 integrin cell surface interaction (20%). Interestingly, the structural constituents of cytoskeleton, ribosome, and extracellular matrix were dominantly enriched in both meniscal tissues by functional analysis.

### 2.3. Development of the MRM Method for ECM Proteins

ECM played a structural role and contributed to the mechanical properties of cartilage tissues. Thus, we focused on 131 ECM-related proteins (130 genes) identified in meniscal tissues by LC-MS/MS analysis. A number of the 131 proteins assigned to ECM proteins was analyzed by the MRM method. Herein, 3024 MRM transitions were generated using Skyline software against 474 peptides from 131 ECM proteins of interest. These MRM transitions were experimentally refined in protein extracts obtained from pooled meniscal tissues. Of them, MRM assays of 119 proteins were established (Supplementary Table S3).

### 2.4. Differentially Expressed ECM Proteins Identified in the Meniscus by MRM Assay

**Figure 1:**
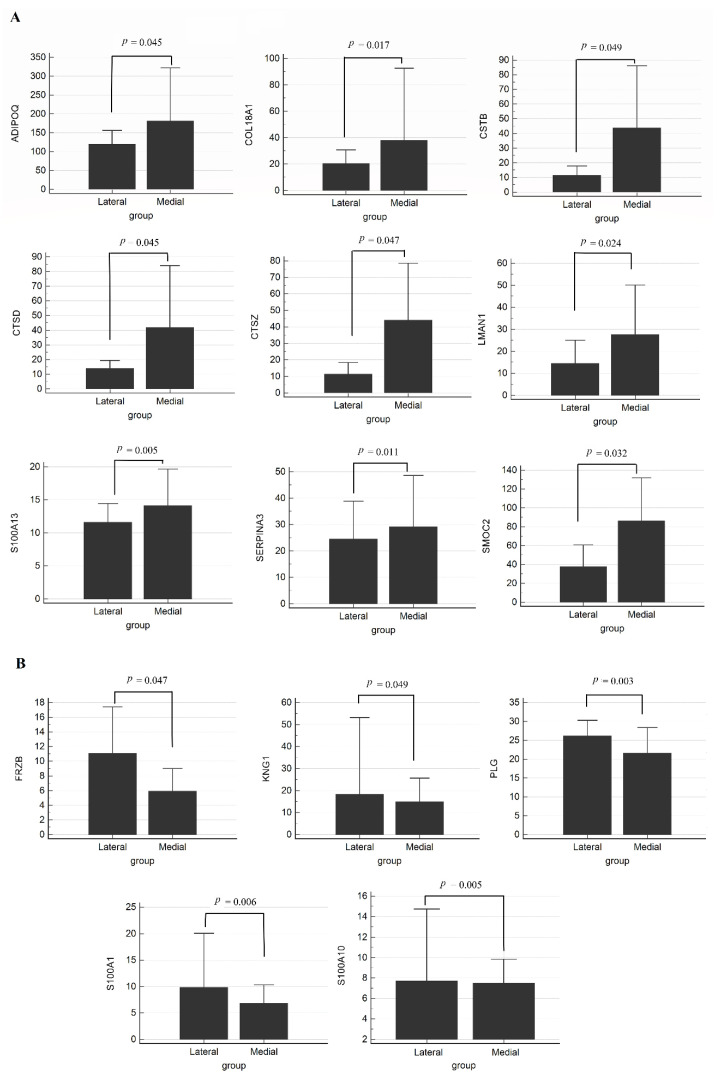
MRM results of meniscal tissues. (**A**) Increased proteins in medial lesions, (**B**) Decreased proteins in medial lesions by the Wilcoxon test. A *p*-value < 0.05 is statistically significant.

To identify ECM proteins differentially expressed in lateral and medial meniscus, we measured 75 ECM proteins of interest in 12 lateral/medial pairs obtained from meniscus tissues from osteoarthritis patients during total knee arthroscopy using MRM assay. Herein, we showed that 14 proteins were different in the two lesions using the Wilcoxon test (*p* < 0.05) (Supplementary Table S4, Figure 1). Nine proteins consisting of collagen alpha-1(XVIII) chain (COL18A1), Cystatin-B (CSTB), Cathepsin D (CTSD), Cathepsin Z (CTSZ), Protein ERGIC-53 (LMAN1), Protein S100-A13 (S100A13), Adiponectin (ADIPOQ), Alpha-1-antichymotrypsin (SERPINA3), and SPARC-related modular calcium-binding protein 2 (SMOC2) had greater expression in the medial lesion compared to the lateral (Figure 1A). In contrast, expression of the five proteins Kininogen-1 (KNG1), Secreted frizzled-related protein 3 (FRZB), Plasminogen (PLG), Protein S100-A1 (S100A1), and Protein S100-A10 (S100A10) decreased in the medial lesions (Figure 1B). Interestingly, among these proteins, the MRM results of SMOC2 and FRZB are consistent with those of the TMT experiment, where SMOC2 increased 2.3-fold and FRZB decreased 2-fold in medial lesions.

## 3. Discussion

To investigate the type and distribution of ECM proteins in meniscal tissue is very important in cartilage tissue engineering as well as in identifying related disease etiologies, including osteoarthritis. Several studies have profiled the proteome of articular cartilage and medial meniscus tissue, but the ECM profile in these tissues is not sufficiently covered and quantified [8,9]. Thus, in this study, we characterized the proteomic profile of meniscal tissues consisting of medial and lateral lesions from osteoarthritis patients, which are still uncovered their protein composition but also quantified. We focused on meniscal ECM proteins, which contribute to repair and regenerate tissues and reported that ECM composition and expression levels in both lesions using both TMT analysis and MRM-MS. Herein we first provided the characterization of osteoarthritic meniscus using quantitative-based proteomic analysis, although there was few concentration change for major cartilage proteins such as COMP, CILP, CAN and collagens in the protein expression profile between lateral and medial meniscus.

Recently only a few pairwise comparisons of the lateral and medial osteoarthritis knee have been conducted (Supplementary Table S5). Moreover, only one study was performed for the proteomic comparison between osteoarthritis menisci. Our study was conducted as a preliminary study to help us understand the mechanisms of osteoarthritis by comparing protein composition within lateral and medial from an expanded number of osteoarthritis subjects. We think it is important to analyze ECM proteins and to discover biomarkers that are definitely lacking in this field.

Osteoarthritis is caused by failure of chondrocytes to maintain homeostasis between synthesis and degradation of ECM components, such as polypeptide, growth factors and cytokines. Recent studies reported that the Wnt/β-catenin pathway plays an important role in the pathophysiology of osteoarthritis [10]. We found that these differentially expressed ECM proteins were increased in regulation of peptidase activity, biological extracellular matrix processes, and ECM modulation. Among these ECM proteins, FRZB showed lower expression in the medial meniscus than lateral meniscus, whereas SMOC2, CSTB, CTSD, and CTSZ showed higher expression in the medial than lateral meniscus. FRZB acts as the WNT inhibitor, and loss of function of FRZB results from excessive WNT activation and increases susceptibility to osteoarthritis [10]. SMOC2 is a member of the secreted protein acidic and rich in cysteine (SPARC) family and is associated with adult wound healing and age-dependent bone loss. Lu et al. recently demonstrated that SMOC2 directly interacted with WNT receptors and activated the WNT/β-catenin pathway in endometrial carcinoma [11]. Furthermore, inflammation modulators (such as CSTB, CTSD, and CTSZ), which have been reported to contribute to osteoarthritis, were more highly expressed in the medial than lateral meniscus [12]. These proteins might play an important role in regulation of chondrocyte growth and proliferation compared to cartilage proteins, which have a slow turnover rate. Therefore, alteration of these proteins can affect ECM regeneration, leading to promotion of osteoarthritis.

Our study has some limitations. First, we could not perform comparisons between osteoarthritis patients and healthy controls, because it is difficult to obtain intact meniscus samples from healthy subjects. Also, it is unclear whether the differences in protein expression levels of ECM in lateral and medial menisci from osteoarthritis patients directly correlate with tissue damage. Further study should be conducted to elucidate the relationship between the patterns of protein expression in meniscus and tissue damage in osteoarthritis.

Despite these limitations, the results of this study would be expected to provide important information in the treatment and diagnosis of various joint diseases as well as osteoarthritis in the future.

## 4. Methods

### 4.1. Clinical Samples

This study enrolled 12 osteoarthritis patients who received knee arthroscopy surgery at Samsung Medical Center. All 12 patients were female and had a mean age of 72.9 years (range 65–82 year). Tissue from lateral/medial menisci pairs was obtained during surgery, and the mean weight of the tissues was 2260 mg (range 950–4100 mg). Written informed consent was obtained from all enrolled patients. This study was approved by the Institutional Review Board (IRB) of Samsung Medical Center (Seoul, Korea) (IRB file No. 2015-12-166).

### 4.2. Preparation of Tissues

**Figure 2:**
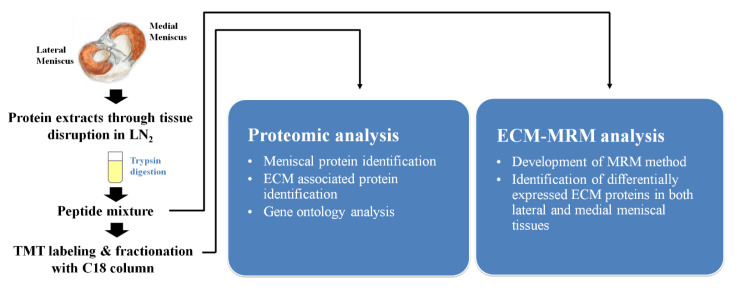
Summary of the experimental design.

About 200–300 mg of cartilage tissue was cut from each sample and pulverized in liquid nitrogen using a ball grinder. RIPA buffer (150 mM sodium chloride, 1% Triton X-100, 1% sodium deoxycholate, 0.1% SDS, 50 mM Tris-HCl pH 7.5, 2 mM EDTA, sterile solution, protease inhibitor) was added to the pulverized tissue and sonicated four times for 15 s each. A total of 500 µg of protein extract was subjected to FASP [13]. Trypsin digestion was performed in a microwave at 450 W and 55 °C for one hour using a Rapid Enzyme Digestion system (Asta, Seoul, Korea). Desalting and concentration of the samples were performed using a StrataTM-X 33 um (Phenomenex Inc., Torrance, CA, USA) according to manufacturer instructions. These protein extracts were subjected to further mass spectrometry-based comparative proteomic analysis and an MRM assay (Figure 2).

### 4.3. LC-MS/MS Analysis

In-solution digestion with trypsin, followed by 6-plex TMT treatment were performed according to the manufacturer’s instructions (Thermo Fisher Scientific, Waltham, MA, USA). After pooling each disease group, labeled peptide mixtures were separated via reverse phase HPLC into 12 fractions and were analyzed using an Orbitrap Elite mass spectrometer (Thermo Finnigan, San Jose, CA, USA) equipped with a nano-electrospray ion source.

The TMT-labeled peptide mixtures were analyzed using an Orbitrap Elite mass spectrometer equipped with a nano-electrospray ion source. The mobile phase consisted of buffer A (0.1% formic acid in water) and buffer B (0.1% formic acid in ACN). After injecting a sample onto the analytical column (75 um × 50 cm packed C18, 2 um particles, 100 Å pores) (Thermo Finnigan, CA, USA), a 90-min gradient method was used to separate the peptide mixture. Sample loading onto the analytical column was conducted at 3% buffer B, the mobile phase was held at 4% buffer B for 1 min, followed by a linear gradient to 32% buffer B over 91 min, followed by a linear gradient to 80% buffer B over 8 min at a flow rate of 300 nL/min.

### 4.4. MRM Assay for ECM Protein Determination

To develop the MRM assay, ECM MatrisomeDB 2.0 depository (http://www.matrisomedb.org/) (accessed on 20 September 2018) was used to extract ECM-related proteins from the identified meniscal tissue profile. Those peptides and MRM transitions were generated using Skyline 4.1.011796 (MacCoss Lab Software, Seattle, WA, USA) and employed for further refinement of the selected peptides. At least three transitions from one proteotypic peptide were generated; 2 or 3 peptide charge states containing 8–30 amino acids, no post-translational modification (PTM), and non-specific cleavage were not allowed. Therefore, a total of 3024 MRM transitions was generated against 131 ECM proteins and 474 peptides. These MRM transitions were refined experimentally in protein extracts obtained from pooled meniscal tissues. A minimum of 3 MRM transitions per peptide should match the same retention times with S/N > 10. MRM was performed in positive mode using a QTRAP 5500 hybrid triple quadrupole/linear ion trap mass spectrometer (Sciex, Framingham, MA, USA) interfaced with a nano-electrospray ion source.

### 4.5. Data Annotation

All MS/MS spectra were searched against the UniProt Human protein database (8 August 2016, Reviewed 20197proteins) using the Integrated Proteomics Pipeline v.3 (IP2) search algorithm for peptide identification. The search parameters were as follows: specific to trypsin with two missed cleavages, variable modification of methionine oxidation, fixed modification of carbamidomethyl cysteine, ±10 ppm precursor-ion tolerance, ±600 ppm fragment-ion tolerance, and ±10 reporter ion tolerance.

Peak area ratio (PAR) was calculated to compare expression profiles among meniscal tissues. The peak area of each peptide transition was divided by the peak area of the corresponding transition from the isotope-labeled peptide. The concentration of these proteins was calculated as the product of PAR.

Gene Ontology (GO) analysis was performed using Funrich software (Version 3.1.3) and the web-based browser STRING (https://string-db.org) (accessed on 19 November 2019) to classify the cellular components, biological process, and molecular function.

### 4.6. Statistics

Data acquired from MRM experiments were analyzed using the SPSS statistical package (IBM Corporation, Somers, NY, USA) ver 21.0 and MedCalc software ver 19.0.7 (Mariakerke, Belgium). Non-parametric tests were used for all proteomic markers. The proteomic differences in expression of markers between lateral and medial menisci groups were assessed with the Wilcoxon test. A *p*-value ≤ 0.05 was considered statistically significant.

## Data Availability

The data presented in this study are openly available in the MassIVE at https://massive.ucsd.edu, (accessed on 19 November 2019), reference number [MSV000087753].

## Supplementary Materials

The following are available online at https://www.mdpi.com/article/10.3390/ijms22158181/s1.

## References

[1] Makris E.A., Hadidi P., Athanasiou K.A. (2011). The knee meniscus: Structure-function, pathophysiology, current repair techniques, and prospects for regeneration. Biomaterials.

[2] Poulsen E., Goncalves G.H., Bricca A., Roos E.M., Thorlund J.B., Juhl C.B. (2019). Knee osteoarthritis risk is increased 4-6 fold after knee injury—A systematic review and meta-analysis. Br. J. Sports Med..

[3] Hsueh M.F., Khabut A., Kjellstrom S., Onnerfjord P., Kraus V.B. (2016). Elucidating the Molecular Composition of Cartilage by Proteomics. J. Proteome Res..

[4] Bell P.A., Wagener R., Zaucke F., Koch M., Selley J., Warwood S., Knight D., Boot-Handford R.P., Thornton D.J., Briggs M.D. (2013). Analysis of the cartilage proteome from three different mouse models of genetic skeletal diseases reveals common and discrete disease signatures. Biol. Open.

[5] Ribitsch I., Mayer R.L., Egerbacher M., Gabner S., Kandula M.M., Rosser J., Haltmayer E., Auer U., Gultekin S., Huber J. (2018). Fetal articular cartilage regeneration versus adult fibrocartilaginous repair: Secretome proteomics unravels molecular mechanisms in an ovine model. Dis. Model. Mech..

[6] Liu S., Zhang W., Zhang F., Roepstorff P., Yang F., Lu Z., Ding W. (2018). TMT-Based Quantitative Proteomics Analysis Reveals Airborne PM2.5-Induced Pulmonary Fibrosis. Int. J. Environ. Res. Public Health.

[7] Svala E., Lofgren M., Sihlbom C., Ruetschi U., Lindahl A., Ekman S., Skioldebrand E. (2015). An inflammatory equine model demonstrates dynamic changes of immune response and cartilage matrix molecule degradation in vitro. Connect. Tissue Res..

[8] Folkesson E., Turkiewicz A., Ryden M., Hughes H.V., Ali N., Tjornstrand J., Onnerfjord P., Englund M. (2020). Proteomic characterization of the normal human medial meniscus body using data-independent acquisition mass spectrometry. J. Orthop. Res..

[9] Folkesson E., Turkiewicz A., Englund M., Onnerfjord P. (2018). Differential protein expression in human knee articular cartilage and medial meniscus using two different proteomic methods: A pilot analysis. BMC Musculoskelet. Disord..

[10] Wang Y., Fan X., Xing L., Tian F. (2019). Wnt signaling: A promising target for osteoarthritis therapy. Cell Commun. Signal..

[11] Lu H., Ju D.D., Yang G.D., Zhu L.Y., Yang X.M., Li J., Song W.W., Wang J.H., Zhang C.C., Zhang Z.G. (2019). Targeting cancer stem cell signature gene SMOC-2 Overcomes chemoresistance and inhibits cell proliferation of endometrial carcinoma. EBioMedicine.

[12] Ben-Aderet L., Merquiol E., Fahham D., Kumar A., Reich E., Ben-Nun Y., Kandel L., Haze A., Liebergall M., Kosińska M.K. (2015). Detecting cathepsin activity in human osteoarthritis via activity-based probes. Arthritis Res. Ther..

[13] Wisniewski J.R., Zougman A., Nagaraj N., Mann M. (2009). Universal sample preparation method for proteome analysis. Nat. Methods.

