# Supplementary material for: Proteomic Analysis of the Meniscus Cartilage in Osteoarthritis
Source: Int J Mol Sci. 2021 Jul 30;22(15):8181. doi: 10.3390/ijms22158181 (PMC8348647; doi:10.3390/ijms22158181)
Supplement: Supplementary file 1 [file ijms-22-08181-s001.zip › Supplementary Table S1.pdf]

Supplementary Table S1. Top 20 proteins dominantly identified in both lateral and medial meniscal tissues by TMT analysis.

Uniprot ID	DESCRIPTION	Gene name	SPEC_COUNT	SEQ_COUNT	Medial to Lateral Ratio (log2)	ECM related Protein
P02768	Serum albumin	ALB	3609	126	-0.08	
P49747	Cartilage oligomeric matrix protein	COMP	857	83	-0.62	v
P08670	Vimentin	VIM	811	63	-0.36	
P02675	Fibrinogen beta chain	FGB	423	49	3.43	
P51888	Prolargin	PRELP	376	42	-0.31	v
P02787	Serotransferrin	TF	318	66	-0.04	
P10909	Clusterin	CLU	311	34	0.01	
P63261	Actin, cytoplasmic 2	ACTG1	277	40	-0.13	
P02751	Fibronectin	FN1	261	85	1.23	v
P01009	Alpha-1-antitrypsin	SERPINA1	240	44	-0.01	v
P01857	Ig gamma-1 chain C region	IGHG1	230	29	0.23	
P12111	Collagen alpha-3(VI) chain	COL6A3	229	105	-0.26	v
O75339	Cartilage intermediate layer protein 1	CILP	215	60	-0.61	v
P01024	Complement C3	C3	210	99	0.07	
P07585	Decorin	DCN	207	35	-0.54	v
Q09666	Neuroblast differentiation-associated protein AHNAK	AHNAK	204	135	-0.24	
P16112	Aggrecan core protein	ACAN	187	39	-0.11	v
P06733	Alpha-enolase	ENO1	184	36	0.30	
P01834	Ig kappa chain C region	IGKC	177	13	0.24	
P68871	Hemoglobin subunit beta	HBB	169	24	1.13	
