# Supplementary material for: Proteomic Analysis of the Meniscus Cartilage in Osteoarthritis
Source: Int J Mol Sci. 2021 Jul 30;22(15):8181. doi: 10.3390/ijms22158181 (PMC8348647; doi:10.3390/ijms22158181)
Supplement: Supplementary file 1 [file ijms-22-08181-s001.zip › Supplementary Table S3.pdf]

Supplementary Table S3. The MRM transitions of 119 ECM proteins

Gene name	Peptide sequnece	Targetion	Light		Heavy		CE
			Q1 Mass (Da)	Q3 Mass (Da)	Q1 Mass (Da)	Q3 Mass (Da)	
		**+2y9**	**868.4**	**1007.5**	**872.4**	**1015.5**	**40.1**
**COL11A2**	**GANEDELSPETSPYVK**	+2y8	868.4	920.5	872.4	928.5	40.1
		+2b8	868.4	816.3	872.4	816.3	40.1
		+2y7	740.4	801.4	744.4	809.4	35.5
**COL12A1**	**DLSFSEVTSYGFK**	**+2y6**	**740.4**	**702.3**	**744.4**	**710.4**	**35.5**
		+2y5	740.4	601.3	744.4	609.3	35.5
		+2y6	465.3	646.4	469.3	654.4	25.6
**COL14A1**	**IGILITDGK**	**+2y5**	**465.3**	**533.3**	**469.3**	**541.3**	**25.6**
		+2y4	465.3	420.2	469.3	428.2	25.6
		**+2y8**	**683.9**	**893.5**	**687.9**	**901.5**	**33.5**
**COL15A1**	**GGVLFAITDAFQK**	+2y7	683.9	822.4	687.9	830.5	33.5
		+2y6	683.9	709.4	687.9	717.4	33.5
		**+2y11**	**801.9**	**1046.6**	**806.9**	**1056.6**	**37.7**
**COL18A1**	**TEAPSATGQASSLLGGR**	+2y10	801.9	945.5	806.9	955.5	37.7
		+2b2	801.9	231.1	806.9	231.1	37.7
		**+2y9**	**545.3**	**805.4**	**550.3**	**815.4**	**28.5**
**COL1A1**	**GVQGPPGPAGPR**	+2y8	545.3	748.4	550.3	758.4	28.5
		+2y7	545.3	651.4	550.3	661.4	28.5
		+2y9	448.2	741.4	452.2	749.4	25
**COL1A2**	**GPAGPSGPAGK**	**+2y8**	**448.2**	**670.4**	**452.2**	**678.4**	**25**
		+2y7	448.2	613.3	452.2	621.3	25
		**+2y12**	**860.4**	**1036.5**	**864.4**	**1044.5**	**39.8**
**COL3A1**	**NGETGPQGPPGPTGPGGDK**	+2y11	860.4	979.5	864.4	987.5	39.8
		+2y7	860.4	631.3	864.4	639.3	39.8
		+2y9	790.9	1077.6	795.9	1087.6	37.3
**COL6A1**	**VFSVAITPDHLEPR**	+2y8	790.9	964.5	795.9	974.5	37.3
		**+2y7**	**790.9**	**863.4**	**795.9**	**873.4**	**37.3**
		**+2y8**	**1097.5**	**862.4**	**735.3**	**872.4**	**48.4**
**COL6A2**	**YGGLHFSDQVEVFSPPGSDR**	+3b8	732.0	877.4	735.3	877.4	37.4
		+3b9	732.0	1005.4	735.3	1005.4	37.4
		**+2y9**	**734.4**	**1056.5**	**739.4**	**1066.5**	**35.3**
**COL6A3**	**ALNLGYALDYAQR**	+2y7	734.4	836.4	739.4	846.4	35.3
		+2y5	734.4	652.3	739.4	662.3	35.3
		+2y9	610.3	971.5	614.3	979.5	30.8
**ABI3BP**	**FTEAIVDAEPK**	+2y6	610.3	658.3	614.3	666.4	30.8
		**+2y2**	**610.3**	**244.2**	**614.3**	**252.2**	**30.8**
**AEBP1**	**YLSPDATVSTEVR**	**+2y10**	**719.4**	**1074.5**	**724.4**	**1084.6**	**34.8**
		+2y7	719.4	791.4	724.4	801.4	34.8

		+2y5	719.4	591.3	724.4	601.3	34.8
		+2y5	595.3	587.3	600.3	597.3	30.3
**CILP**	**LFNLDVPESR**	**+2y4**	**595.3**	**488.2**	**600.3**	**498.3**	**30.3**
		+2y2	595.3	262.2	600.3	272.2	30.3
		+2y8	652.3	816.4	657.3	826.4	32.3
**CILP2**	**DLTSAASAPSDLR**	+2y7	652.3	745.4	657.3	755.4	32.3
		**+2y5**	**652.3**	**587.3**	**657.3**	**597.3**	**32.3**
		**+2y9**	**807.4**	**992.5**	**812.4**	**1002.5**	**37.9**
**COMP**	**NALWHTGDTESQVR**	+2y8	807.4	891.4	812.4	901.4	37.9
		+2y6	807.4	719.4	812.4	729.4	37.9
		+2y7	643.3	898.4	648.3	908.5	32
**EFEMP1**	**SGNENGEFYLR**	**+2y6**	**643.3**	**784.4**	**648.3**	**794.4**	**32**
		+2y4	643.3	598.3	648.3	608.3	32
		+2y7	589.8	857.4	594.8	867.4	30.1
**FBLN1**	**TGYYFDGISR**	**+2y6**	**589.8**	**694.4**	**594.8**	**704.4**	**30.1**
		+2y5	589.8	547.3	594.8	557.3	30.1
		**+2y6**	**565.2**	**700.3**	**570.3**	**710.4**	**29.2**
**FBLN2**	**GNEEGYFGTR**	+2y4	565.2	480.3	570.3	490.3	29.2
		+2y3	565.2	333.2	570.3	343.2	29.2
		+2y9	662.3	932.5	666.3	940.5	32.7
**FBN1**	**DYLSGELGDNLK**	+2y8	662.3	845.4	666.3	853.5	32.7
		**+2b2**	**662.3**	**279.1**	**666.3**	**279.1**	**32.7**
		+2y9	797.4	973.5	801.4	981.5	37.6
**FGA**	**HPDEAAFFDTASTGK**	+2y6	797.4	564.3	801.4	572.3	37.6
		**+2b2**	**797.4**	**235.1**	**801.4**	**235.1**	**37.6**
		+2y8	768.4	903.5	772.4	911.5	36.5
**FGB**	**AHYGGFTVQNEANK**	+2y6	768.4	703.3	772.4	711.4	36.5
		**+2b3**	**768.4**	**372.2**	**772.4**	**372.2**	**36.5**
		+2y8	746.4	928.5	751.4	938.5	35.7
**FGG**	**YEASILTHDSSIR**	**+2y7**	**746.4**	**815.4**	**751.4**	**825.4**	**35.7**
		+2y6	746.4	714.4	751.4	724.4	35.7
		+2y8	678.4	966.5	683.4	976.5	33.3
**FN1**	**IYLYTLNDNAR**	**+2y7**	**678.4**	**803.4**	**683.4**	**813.4**	**33.3**
		+2y5	678.4	589.3	683.4	599.3	33.3
		**+2y10**	**927.4**	**974.5**	**932.4**	**984.5**	**42.2**
**FNDC1**	**TPHSGAAEEDSSASAPPSR**	+2b10	927.4	995.4	932.4	995.4	42.2
		+2b11	927.4	1082.4	932.4	1082.4	42.2
		+2y8	657.8	868.5	662.8	878.5	32.5
**IGFALS**	**DFALQNPSAVPR**	+2y7	657.8	740.4	662.8	750.4	32.5
		**+2y6**	**657.8**	**626.4**	**662.8**	**636.4**	**32.5**
**LAMC1**	**NTIEETGNLAEQAR**	**+2y8**	**773.4**	**858.4**	**778.4**	**868.5**	**36.7**
		+2y5	773.4	574.3	778.4	584.3	36.7

		+2y4	773.4	503.3	778.4	513.3	36.7
		**+2y8**	**495.3**	**819.4**	**500.3**	**829.5**	**26.7**
**LRG1**	**VAAGAFQGLR**	+2y7	495.3	748.4	500.3	758.4	26.7
		+2y5	495.3	620.4	500.3	630.4	26.7
		+2y8	510.3	774.4	515.3	784.4	27.2
**LTBP2**	**SSAAGEGTLAR**	**+2y7**	**510.3**	**703.4**	**515.3**	**713.4**	**27.2**
		+2y3	510.3	359.2	515.3	369.2	27.2
		+2y8	582.8	895.5	586.8	903.5	29.8
**MATN2**	**VGLLQYGSTVK**	**+2y7**	**582.8**	**782.4**	**586.8**	**790.4**	**29.8**
		+2y6	582.8	654.3	586.8	662.4	29.8
		**+2y9**	**1043.5**	**1062.6**	**1048.5**	**1072.6**	**46.4**
**MFGE8**	**NAVHVNLFETPVEAQYVR**	+2y8	1043.5	961.5	1048.5	971.5	46.4
		+2b9	1043.5	1024.5	1048.5	1024.5	46.4
		+2y6	638.3	728.4	643.3	738.4	31.8
**MGP**	**NANTFISPQQR**	**+2y5**	**638.3**	**615.3**	**643.3**	**625.3**	**31.8**
		+2y4	638.3	528.3	643.3	538.3	31.8
		+2y7	547.8	908.5	551.8	916.5	28.6
**MXRA5**	**WFLEWDAK**	+2y6	547.8	761.4	551.8	769.4	28.6
		**+2y5**	**547.8**	**648.3**	**551.8**	**656.3**	**28.6**
		**+2y10**	**829.4**	**967.5**	**834.4**	**977.5**	**38.7**
**PCOLCE**	**YDALEVFAGSGTSGQR**	+2y9	829.4	820.4	834.4	830.4	38.7
		+2y8	829.4	749.4	834.4	759.4	38.7
		**+2y8**	**917.9**	**1022.5**	**921.9**	**1030.5**	**41.9**
**POSTN**	**FIEGGDGHLFEDEEIK**	+2y7	917.9	909.4	921.9	917.4	41.9
		+2b8	917.9	813.4	921.9	813.4	41.9
		**+2y8**	**772.4**	**968.5**	**777.4**	**978.5**	**36.7**
**SMOC1**	**FSEPDPSHTLEER**	+2y2	772.4	304.2	777.4	314.2	36.7
		+2b5	772.4	576.2	777.4	576.2	36.7
		**+2y8**	**755.4**	**968.5**	**760.4**	**978.5**	**36.1**
**SMOC2**	**LSEPDPSHTLEER**	+2y2	755.4	304.2	760.4	314.2	36.1
		+2b5	755.4	542.2	760.4	542.2	36.1
		**+2y8**	**648.4**	**843.4**	**653.4**	**853.4**	**32.2**
**SRPX2**	**LLIISAPDPSNR**	+2y7	648.4	756.4	653.4	766.4	32.2
		+2y6	648.4	685.3	653.4	695.3	32.2
		+2y7	540.3	851.5	544.3	859.5	28.3
**TGFBI**	**DLLNNHILK**	**+2y6**	**540.3**	**738.4**	**544.3**	**746.4**	**28.3**
		+2b8	540.3	933.5	544.3	933.5	28.3
		+2y9	651.9	960.5	655.9	968.6	32.3
**THBS1**	**AGTLDLSLTVQGK**	**+2y7**	**651.9**	**732.4**	**655.9**	**740.4**	**32.3**
		+2y5	651.9	532.3	655.9	540.3	32.3
**THBS2**	**GTTQIDPNWVIR**	+2y8	700.4	1012.6	705.4	1022.6	34.1
		+2y7	700.4	899.5	705.4	909.5	34.1

		**+2y6**	**700.4**	**784.4**	**705.4**	**794.5**	**34.1**
		+2y7	623.8	893.5	627.8	901.5	31.3
**THBS4**	**KPQDFLEELK**	+2y4	623.8	518.3	627.8	526.3	31.3
		**+2b8**	**623.8**	**987.5**	**627.8**	**987.5**	**31.3**
		+2y7	967.5	860.5	971.5	868.5	43.7
**TNC**	**DLQVSETAETSLTLLWK**	**+2y6**	**967.5**	**773.5**	**971.5**	**781.5**	**43.7**
		+2y3	967.5	446.3	971.5	454.3	43.7
		**+2y7**	**607.8**	**786.4**	**612.8**	**796.4**	**30.7**
**TNXB**	**EVTVQGLEPSR**	+2y4	607.8	488.2	612.8	498.3	30.7
		+2y3	607.8	359.2	612.8	369.2	30.7
		**+2y10**	**823.9**	**1076.5**	**828.9**	**1086.5**	**38.5**
**VTN**	**DVWGIEGPIDAAFTR**	+2y9	823.9	947.5	828.9	957.5	38.5
		+2y8	823.9	890.5	828.9	900.5	38.5
		**+2y8**	**584.8**	**926.5**	**589.8**	**936.5**	**29.9**
**ACAN**	**LEGEVFFATR**	+2y6	584.8	740.4	589.8	750.4	29.9
		+2y5	584.8	641.3	589.8	651.3	29.9
		+2y9	990.1	1010.6	994.1	1018.6	44.5
**ASPN**	**LYLSHNQLSEIPLNLPK**	**+2y6**	**990.1**	**681.4**	**994.1**	**689.4**	**44.5**
		+2b8	990.1	969.5	994.1	969.5	44.5
		+2y9	791.5	1033.6	795.5	1041.6	37.3
**BGN**	**GLQHLYALVLVNNK**	**+2b5**	**791.5**	**549.3**	**795.5**	**549.3**	**37.3**
		+2b9	791.5	995.6	795.5	995.6	37.3
		**+2y10**	**683.4**	**1010.5**	**687.4**	**1018.5**	**33.5**
**CHAD**	**NQLSSYPSAALSK**	+2y9	683.4	923.5	687.4	931.5	33.5
		+2y7	683.4	673.4	687.4	681.4	33.5
		**+2y8**	**624.9**	**884.6**	**628.9**	**892.6**	**31.4**
**DCN**	**NLHALILVNNK**	+2y7	624.9	813.5	628.9	821.5	31.4
		+2y6	624.9	700.4	628.9	708.4	31.4
		+2y7	629.8	869.4	634.8	879.4	31.5
**FMOD**	**LYLDHNNLTR**	**+2y6**	**629.8**	**754.4**	**634.8**	**764.4**	**31.5**
		+2y5	629.8	617.3	634.8	627.3	31.5
		+2y8	565.3	816.4	569.3	824.5	29.2
**HAPLN1**	**DPTAFGSGIHK**	**+2y7**	**565.3**	**745.4**	**569.3**	**753.4**	**29.2**
		+2y6	565.3	598.3	569.3	606.3	29.2
		**+2y6**	**584.3**	**741.4**	**589.3**	**751.4**	**29.9**
**HSPG2**	**AFAHLQVPER**	+2y5	584.3	628.3	589.3	638.4	29.9
		+2b7	584.3	767.4	589.3	767.4	29.9
		+2y7	649.3	853.5	653.3	861.5	32.2
**LUM**	**SLEDLQLTHNK**	+2y5	649.3	612.3	653.3	620.4	32.2
		**+2y4**	**649.3**	**499.3**	**653.3**	**507.3**	**32.2**
**OGN**	**LSLLEELSLAENQLLK**	**+2y9**	**907.0**	**1015.6**	**911.0**	**1023.6**	**41.5**
		+2y7	907.0	815.5	911.0	823.5	41.5

		+2y6	907.0	744.4	911.0	752.4	41.5
		+2y8	575.3	923.5	579.3	931.5	29.6
**OMD**	**LLLGYNEISK**	**+2y7**	**575.3**	**810.4**	**579.3**	**818.4**	**29.6**
		+2y6	575.3	753.4	579.3	761.4	29.6
		+2y9	830.9	1042.6	834.9	1050.6	38.8
**PRELP**	**LSDGVFKPDTFHGLK**	+2y8	830.9	914.5	834.9	922.5	38.8
		**+2y6**	**830.9**	**702.4**	**834.9**	**710.4**	**38.8**
		+2y6	826.0	699.4	831.0	709.4	38.6
**PRG4**	**GLPNVVTSAISLPNIR**	+2y5	826.0	612.4	831.0	622.4	38.6
		**+2y4**	**826.0**	**499.3**	**831.0**	**509.3**	**38.6**
		**+2y8**	**657.9**	**930.5**	**662.9**	**940.5**	**32.5**
**VCAN**	**LATVGELQAAWR**	+2y4	657.9	503.3	662.9	513.3	32.5
		+2y2	657.9	361.2	662.9	371.2	32.5
		**+2y9**	**802.9**	**978.5**	**806.9**	**986.5**	**37.8**
**A2M**	**IAQWQSFQLEGGLK**	+2y4	802.9	374.2	806.9	382.3	37.8
		+2b3	802.9	313.2	806.9	313.2	37.8
		+2y9	634.9	956.6	638.9	964.6	31.7
**AGT**	**ALQDQLVLVAAK**	**+2y3**	**634.9**	**289.2**	**638.9**	**297.2**	**31.7**
		+2b9	634.9	980.6	638.9	980.6	31.7
		+2y4	511.3	565.3	516.3	575.3	27.3
**AMBP**	**ETLLQDFR**	+2y3	511.3	437.2	516.3	447.2	27.3
		**+2y2**	**511.3**	**322.2**	**516.3**	**332.2**	**27.3**
		+2y6	927.5	708.4	932.5	718.4	42.2
**CD109**	**SPVTLTAYIVTSLLGYR**	**+2b2**	**927.5**	**185.1**	**932.5**	**185.1**	**42.2**
		+2b3	927.5	284.2	932.5	284.2	42.2
		**+2y9**	**663.9**	**1012.5**	**667.9**	**1020.6**	**32.8**
**CSTB**	**SQVVAGTNYFIK**	+2y8	663.9	913.5	667.9	921.5	32.8
		+2y7	663.9	842.4	667.9	850.5	32.8
		+2y8	521.8	856.6	525.8	864.6	27.6
**CTSD**	**VSTLPAITLK**	**+2y6**	**521.8**	**642.4**	**525.8**	**650.4**	**27.6**
		+2b2	521.8	187.1	525.8	187.1	27.6
		+2y7	609.3	830.4	614.3	840.4	30.8
**CTSZ**	**NSWGEPWGER**	**+2y5**	**609.3**	**644.3**	**614.3**	**654.3**	**30.8**
		+2b3	609.3	388.2	614.3	388.2	30.8
		**+2y8**	**1015.0**	**1055.6**	**1020.0**	**1065.6**	**45.4**
**F12**	**NKPGVYTDVAYYLAWIR**	+2y7	1015.0	984.5	1020.0	994.5	45.4
		+2b8	1015.0	875.4	1020.0	875.4	45.4
		+2y10	846.4	1083.5	850.4	1091.6	39.3
**F13A1**	**DGTHVVENVDATHIGK**	+2y6	846.4	626.4	850.4	634.4	39.3
		**+2b5**	**846.4**	**510.2**	**850.4**	**510.2**	**39.3**
**F2**	**TATSEYQTFFNPR**	**+2y8**	**781.4**	**1072.5**	**786.4**	**1082.5**	**37**
		+2y7	781.4	909.5	786.4	919.5	37

		+2y4	781.4	533.3	786.4	543.3	37
		**+2y9**	**912.9**	**1001.5**	**917.0**	**1009.5**	**41.7**
**HRG**	**ADLFYDVEALDLESPK**	+2y6	912.9	688.4	917.0	696.4	41.7
		+2y3	912.9	331.2	917.0	339.2	41.7
		+2y8	599.3	920.5	603.3	928.5	30.4
**HTRA1**	**YNFIADVVEK**	+2y6	599.3	660.4	603.3	668.4	30.4
		**+2b2**	**599.3**	**278.1**	**603.3**	**278.1**	**30.4**
		+2y9	1003.5	973.5	1008.5	983.5	45
**ITIH1**	**FAHYVVTSQVVNTANEAR**	+2y8	1003.5	874.4	1008.5	884.4	45
		**+2y7**	**1003.5**	**775.4**	**1008.5**	**785.4**	**45**
		**+2y8**	**902.9**	**1019.5**	**907.9**	**1029.5**	**41.4**
**ITIH2**	**AEDHFSVIDFNQNIR**	+2y6	902.9	791.4	907.9	801.4	41.4
		+2b7	902.9	786.3	907.9	786.3	41.4
		+2y5	937.9	697.4	942.9	707.4	42.6
**KNG1**	**YNSQNQSNNQFVLYR**	+2y4	937.9	550.3	942.9	560.3	42.6
		**+2y3**	**937.9**	**451.3**	**942.9**	**461.3**	**42.6**
		**+2y8**	**687.9**	**919.5**	**692.9**	**929.5**	**33.6**
**MMP2**	**QDIVFDGIAQIR**	+2y6	687.9	657.4	692.9	667.4	33.6
		+2y4	687.9	487.3	692.9	497.3	33.6
		+2y8	597.8	966.5	602.8	976.5	30.4
**MMP3**	**LDSDTLEVM[Oxi]R**	+2y5	597.8	663.3	602.8	673.4	30.4
		**+2y4**	**597.8**	**550.3**	**602.8**	**560.3**	**30.4**
		**+2y8**	**678.3**	**1045.5**	**683.3**	**1055.5**	**33.3**
**P4HA2**	**YFEQLLEEER**	+2y4	678.3	562.2	683.3	572.3	33.3
		+2y3	678.3	433.2	683.3	443.2	33.3
		**+2y9**	**649.8**	**1074.6**	**654.8**	**1084.6**	**32.3**
**PLG**	**HSIFTPETNPR**	+2y8	649.8	961.5	654.8	971.5	32.3
		+2y6	649.8	713.4	654.8	723.4	32.3
		**+2y9**	**1029.5**	**1010.4**	**1033.5**	**1018.5**	**45.9**
**SERPINA1**	**LYHSEAFTVNFGDTEEAK**	+2y5	1029.5	577.3	1033.5	585.3	45.9
		+2b7	1029.5	848.4	1033.5	848.4	45.9
		**+2y11**	**954.5**	**1005.5**	**958.5**	**1013.5**	**43.2**
**SERPINA3**	**AVLDVFEEGTEASAATAVK**	+2y8	954.5	718.4	958.5	726.4	43.2
		+2b8	954.5	903.4	958.5	903.4	43.2
		+2y9	596.8	1008.5	601.8	1018.5	30.3
**SERPINA4**	**IAPANADFAFR**	+2y8	596.8	911.4	601.8	921.4	30.3
		**+2y7**	**596.8**	**840.4**	**601.8**	**850.4**	**30.3**
		+2y8	581.3	892.4	586.3	902.4	29.8
**SERPINA5**	**AVVEVDESGTR**	**+2y7**	**581.3**	**763.4**	**586.3**	**773.4**	**29.8**
		+2y6	581.3	664.3	586.3	674.3	29.8
**SERPINE2**	**DIVTVANAVFVK**	**+2y7**	**638.4**	**748.4**	**642.4**	**756.4**	**31.8**
		+2y3	638.4	393.3	642.4	401.3	31.8

		+2b9	638.4	883.5	642.4	883.5	31.8
		+2y5	607.8	544.3	611.8	552.3	30.7
**SERPINF1**	**ELLDTVTAPQK**	+2y4	607.8	443.3	611.8	451.3	30.7
		**+2y3**	**607.8**	**372.2**	**611.8**	**380.2**	**30.7**
		+2y8	977.0	870.6	981.0	878.6	44
**SERPINF2**	**QLTSGPNQEQVSPLTLLK**	**+2y7**	**977.0**	**771.5**	**981.0**	**779.5**	**44**
		+2y6	977.0	684.5	981.0	692.5	44
		**+2y9**	**914.0**	**1055.6**	**919.0**	**1065.6**	**41.8**
**SERPING1**	**GVTSVSQIFHSPDLAIR**	+2y7	914.0	771.4	919.0	781.4	41.8
		+2y4	914.0	472.3	919.0	482.3	41.8
		+2y7	612.8	840.5	616.8	848.5	30.9
**SERPINH1**	**GVVEVTHDLQK**	**+2y6**	**612.8**	**741.4**	**616.8**	**749.4**	**30.9**
		+2b10	612.8	1078.6	616.8	1078.6	30.9
		+2y9	617.3	901.5	622.3	911.5	31.1
**TIMP1**	**GFQALGDAADIR**	+2y8	617.3	830.4	622.3	840.4	31.1
		**+2y7**	**617.3**	**717.4**	**622.3**	**727.4**	**31.1**
		+2y9	803.5	1013.6	807.5	1021.6	37.8
**ANXA1**	**ALTGHLEEVVLALLK**	+2b6	803.5	593.3	807.5	593.3	37.8
		**+2b10**	**803.5**	**1049.6**	**807.5**	**1049.6**	**37.8**
		+2y9	843.4	957.4	848.4	967.4	39.2
**ANXA11**	**SLYHDISGDTSGDYR**	+2y6	843.4	698.3	848.4	708.3	39.2
		**+2y2**	**843.4**	**338.2**	**848.4**	**348.2**	**39.2**
		**+2y6**	**556.3**	**755.4**	**561.3**	**765.4**	**28.9**
**ANXA2**	**QDIAFAYQR**	+2y5	556.3	684.3	561.3	694.4	28.9
		+2y3	556.3	466.2	561.3	476.2	28.9
		+2y9	833.9	971.6	837.9	979.6	38.9
**ANXA4**	**SETSGSFEDALLAIVK**	+2y5	833.9	543.4	837.9	551.4	38.9
		**+2y4**	**833.9**	**430.3**	**837.9**	**438.3**	**38.9**
		+2y9	853.0	1003.6	858.0	1013.6	39.6
**ANXA5**	**GLGTDEESILTLLTSR**	**+2y7**	**853.0**	**803.5**	**858.0**	**813.5**	**39.6**
		+2y6	853.0	690.4	858.0	700.4	39.6
		**+2y7**	**588.8**	**744.5**	**592.8**	**752.5**	**30.1**
**ANXA6**	**DAFVAIVQSVK**	+2y5	588.8	560.3	592.8	568.4	30.1
		+2b4	588.8	433.2	592.8	433.2	30.1
		+2y9	845.9	956.6	850.9	966.6	39.3
**ANXA7**	**GFGTDEQAIVDVVANR**	**+2y7**	**845.9**	**772.4**	**850.9**	**782.4**	**39.3**
		+2y6	845.9	673.4	850.9	683.4	39.3
		+2b2	964.5	262.1	968.5	262.1	43.6
**C1QC**	**FNAVLTNPQGDYDTSTGK**	**+2b3**	**964.5**	**333.2**	**968.5**	**333.2**	**43.6**
		+2b4	964.5	432.2	968.5	432.2	43.6
**CLEC11A**	**DAVQALQEAQGR**	**+2y6**	**643.3**	**688.3**	**648.3**	**698.3**	**32**
		+2b9	643.3	926.5	648.3	926.5	32

		+2b10	643.3	1054.5	648.3	1054.5	32
		**+2y8**	**933.5**	**1050.5**	**938.5**	**1060.5**	**42.5**
**CLEC3A**	**FVDVNGIAISFLNWDR**	+2y5	933.5	703.4	938.5	713.4	42.5
		+2y4	933.5	590.3	938.5	600.3	42.5
		**+2y8**	**657.4**	**871.5**	**661.4**	**879.5**	**32.5**
**CLEC3B**	**LDTLAQEVALLK**	+2y7	657.4	800.5	661.4	808.5	32.5
		+2b3	657.4	330.2	661.4	330.2	32.5
		+2y7	965.5	742.4	970.5	752.4	43.6
**CSPG4**	**ALLHVWAGGPWPQGATLR**	**+2b5**	**965.5**	**534.3**	**970.5**	**534.3**	**43.6**
		+2b7	965.5	791.5	970.5	791.5	43.6
		+2y7	571.8	820.5	575.8	828.5	29.4
**HPX**	**QGHNSVFLIK**	+2y6	571.8	706.5	575.8	714.5	29.4
		**+2y2**	**571.8**	**260.2**	**575.8**	**268.2**	**29.4**
		+2y6	538.7	776.4	543.7	786.4	28.3
**LGALS1**	**DGGAWGTEQR**	**+2y5**	**538.7**	**590.3**	**543.7**	**600.3**	**28.3**
		+2b5	538.7	487.2	543.7	487.2	28.3
		**+2y6**	**486.8**	**744.4**	**491.8**	**754.4**	**26.4**
**LMAN1**	**DIDNLVQR**	+2y3	486.8	402.2	491.8	412.3	26.4
		+2b5	486.8	571.3	491.8	571.3	26.4
		**+2y9**	**1092.6**	**903.5**	**1096.6**	**911.5**	**48.2**
**ANGPTL2**	**QIETLQQLVEVDGGIVSEVK**	+2y8	1092.6	788.5	1096.6	796.5	48.2
		+2b9	1092.6	1053.6	1096.6	1053.6	48.2
		+2y5	464.7	671.4	469.8	681.4	25.6
**ANGPTL7**	**SGLVSFYR**	**+2y4**	**464.7**	**572.3**	**469.8**	**582.3**	**25.6**
		+2y3	464.7	485.3	469.8	495.3	25.6
		**+2y9**	**819.4**	**1078.5**	**824.4**	**1088.5**	**38.4**
**FGFBP2**	**QGSTGEEFHFQTGGR**	+2y8	819.4	949.5	824.4	959.5	38.4
		+2b9	819.4	973.4	824.4	973.4	38.4
		+2y9	586.4	945.5	590.4	953.5	30
**FRZB**	**LLLVEGSIAEK**	+2y8	586.4	832.4	590.4	840.5	30
		**+2y7**	**586.4**	**733.4**	**590.4**	**741.4**	**30**
		+2y6	926.9	674.4	931.0	682.4	42.2
**MST1**	**WDLQHPHQHPFEPGK**	**+2y3**	**926.9**	**301.2**	**931.0**	**309.2**	**42.2**
		+2b5	926.9	680.3	931.0	680.3	42.2
		+2y9	846.4	978.5	850.5	986.5	39.3
**S100A1**	**ELLQTELSGFLDAQK**	+2y8	846.4	865.4	850.5	873.5	39.3
		**+2b2**	**846.4**	**243.1**	**850.5**	**243.1**	**39.3**
		**+2y8**	**604.8**	**932.5**	**608.8**	**940.5**	**30.6**
**S100A10**	**EFPGFLENQK**	+2y5	604.8	631.3	608.8	639.4	30.6
		+2y4	604.8	518.3	608.8	526.3	30.6
**S100A11**	**DGYNYTLSK**	**+2y6**	**530.8**	**725.4**	**534.8**	**733.4**	**28**
		+2y5	530.8	611.3	534.8	619.4	28

		+2y4	530.8	448.3	534.8	456.3	28
		+2y9	624.3	1047.5	628.3	1055.5	31.3
**S100A13**	**SLDVNQDSELK**	+2y7	624.3	833.4	628.3	841.4	31.3
		**+2b3**	**624.3**	**316.2**	**628.3**	**316.2**	**31.3**
		+2y6	455.2	693.4	459.2	701.4	25.2
**S100A4**	**TDEAAFQK**	+2y5	455.2	564.3	459.2	572.3	25.2
		**+2b2**	**455.2**	**217.1**	**459.2**	**217.1**	**25.2**
		**+2y6**	**458.2**	**674.3**	**463.3**	**684.4**	**25.4**
**S100A6**	**LQDAEIAR**	+2y5	458.2	559.3	463.3	569.3	25.4
		+2b2	458.2	242.2	463.3	242.2	25.4
		+2y5	482.2	645.3	487.2	655.4	26.2
**S100A8**	**GNFHAVYR**	**+2y4**	**482.2**	**508.3**	**487.2**	**518.3**	**26.2**
		+2y3	482.2	437.3	487.2	447.3	26.2
		+2y7	459.7	755.4	464.7	765.4	25.4
**SCUBE1**	**YALHSDGR**	+2y6	459.7	684.3	464.7	694.4	25.4
		**+2y5**	**459.7**	**571.3**	**464.7**	**581.3**	**25.4**
		**+2y8**	**587.3**	**958.6**	**591.3**	**966.6**	**30**
**SFRP1**	**SQYLLTAIHK**	+2y7	587.3	795.5	591.3	803.5	30
		+2y6	587.3	682.4	591.3	690.4	30
		+2y9	706.3	839.4	711.3	849.4	34.3
**ADIPOQ**	**GDIGETGVPGAEGPR**	**+2y7**	**706.3**	**683.3**	**711.3**	**693.4**	**34.3**
		+2b8	706.3	729.3	711.3	729.3	34.3
		+2y7	570.3	809.4	575.3	819.4	29.4
**DPT**	**GATTTFSAVER**	**+2y6**	**570.3**	**708.4**	**575.3**	**718.4**	**29.4**
		+2y5	570.3	561.3	575.3	571.3	29.4

NOTE: Endogenous peptides (light) and isotope-labeled peptides (heavy) were chosen to measure the target proteins of interest. C-terminal peptides (arginine [R] or lysine [K]) were labeled with the 13C15N heavy isotope. Quantitative ion is marked in bold font.
