# Supplementary material for: Proteomic Analysis of the Meniscus Cartilage in Osteoarthritis
Source: Int J Mol Sci. 2021 Jul 30;22(15):8181. doi: 10.3390/ijms22158181 (PMC8348647; doi:10.3390/ijms22158181)
Supplement: Supplementary file 1 [file ijms-22-08181-s001.zip › Supplementary Table S4.pdf]

Supplementary Table S4. The statistics results of MRM results.

Gene Name	Peak Area Ratios						*P*-values
	Lateral M. (n=12)			Medial M. (n=12)			
	Median	95% CI	SD	Median	95% CI	SD	**L vs. M**
A2M	4.0	3.435 to 6.516	58.3	11.7	5.033 to 18.811	81.0	0.339
ABI3BP	24.5	19.539 to 45.522	83.1	26.3	20.446 to 51.619	19.4	0.720
ACAN	219.5	112.859 to 356.003	133.7	185.2	114.707 to 351.088	142.5	0.346
ADIPOQ	119.3	98.197 to 156.065	49.9	181.0	106.322 to 321.616	133.6	0.045
AEBP1	19.6	16.907 to 24.948	62.4	18.0	14.017 to 25.151	8.3	0.206
AGT	3.8	3.300 to 4.900	8.7	11.7	4.612 to 18.461	79.8	0.072
AMBP	3.9	3.069 to 5.065	23.2	11.7	5.236 to 17.678	82.7	0.370
ANGPTL2	27.7	11.617 to 47.564	20.4	34.5	15.717 to 60.003	24.0	0.208
ANGPTL7	12.7	7.390 to 18.448	27.5	20.9	9.852 to 47.977	20.9	0.352
ANXA1	84.5	56.885 to 115.642	35.8	110.1	47.493 to 162.478	61.9	0.592
ANXA2	153.5	105.100 to 188.043	51.5	143.7	120.415 to 216.994	116.2	0.753
ANXA4	271.2	210.798 to 354.665	82.2	313.5	100.866 to 463.536	185.5	0.722
ANXA5	143.6	105.154 to 163.748	596.8	155.6	114.050 to 240.632	119.9	0.959
ANXA6	6.0	4.317 to 9.214	78.8	11.0	6.097 to 14.393	4.3	0.666
ANXA7	1.9	1.335 to 4.031	2.8	4.1	2.537 to 5.268	1.8	0.157
ASPN	187.5	68.271 to 329.361	142.9	184.6	110.091 to 365.012	145.4	0.937
BGN	72.9	42.548 to 99.993	32.6	77.6	49.813 to 189.733	186.8	0.119
C1QC	11.4	6.767 to 21.815	25.6	26.6	15.668 to 44.455	14.8	0.153
CHAD	70.9	48.125 to 103.898	49.6	75.8	56.200 to 179.391	182.8	0.286
CILP	22.1	19.986 to 26.547	39.3	17.7	14.442 to 30.324	9.7	0.317
CLEC3B	6.6	5.735 to 11.214	46.8	10.5	6.886 to 14.220	3.8	0.414
COL14A1	21.1	13.321 to 28.324	20.6	4.2	2.100 to 56.034	41.3	0.559
COL15A1	17.6	12.673 to 35.252	50.6	3.1	1.517 to 50.606	40.9	0.469
COL18A1	20.4	11.104 to 30.744	24.0	37.9	2.335 to 92.531	52.3	0.017
COL6A1	22.5	17.225 to 29.354	14.9	26.4	19.446 to 49.470	19.8	0.587

COL6A3	22.3	17.839 to 43.286	23.2	24.7	20.101 to 52.620	19.7	0.858
COMP	23.5	19.032 to 25.962	199.8	20.0	14.286 to 32.530	10.7	0.589
CSTB	11.5	9.238 to 17.796	31.1	43.7	15.664 to 85.994	252.9	0.046
CTSD	14.0	9.415 to 19.283	36.7	41.9	15.588 to 83.927	263.0	0.045
CTSZ	11.3	9.535 to 18.375	34.2	44.0	16.302 to 78.582	249.8	0.047
DPT	146.4	119.025 to 192.753	43.5	203.5	111.939 to 351.656	137.6	0.116
F2	8.4	4.504 to 17.764	8.6	25.6	5.221 to 90.277	80.2	0.059
FBLN1	10.7	6.176 to 18.185	36.3	9.2	5.707 to 11.762	3.8	0.084
FBLN2	6.6	4.604 to 13.827	6.0	8.3	5.317 to 10.631	3.7	0.705
FGB	7.7	5.804 to 16.044	7.1	10.0	5.756 to 13.331	4.6	0.206
FGG	10.8	4.456 to 19.916	10.8	13.4	7.053 to 17.765	7.4	0.584
FN1	15.7	5.804 to 25.993	14.3	15.8	7.522 to 18.062	7.1	0.327
FRZB	11.1	2.218 to 17.393	23.8	5.9	2.638 to 9.014	4.3	0.047
HPX	1.6	0.752 to 6.282	7.1	5.9	2.769 to 9.549	4.0	0.102
HRG	6.7	1.856 to 21.384	9.6	32.6	4.920 to 112.970	91.9	0.176
HTRA1	15.6	4.206 to 32.479	21.5	60.6	10.333 to 114.638	87.7	0.083
ITIH1	16.1	12.273 to 31.432	18.8	16.2	12.567 to 26.796	10.6	0.854
KNG1	18.2	11.652 to 53.189	34.4	15.0	12.859 to 25.579	10.8	0.049
LGALS1	12.8	10.842 to 22.330	15.2	16.9	15.686 to 23.083	6.4	0.426
LMAN1	14.5	8.535 to 25.020	9.9	27.5	10.406 to 50.093	19.4	0.024
LTBP2	22.7	8.559 to 39.098	26.0	19.8	12.784 to 26.013	9.1	0.151
LUM	69.2	41.110 to 95.044	30.6	71.2	47.080 to 175.816	165.2	0.195
MATN2	51.6	24.883 to 95.079	64.3	62.7	23.782 to 74.583	29.9	1.000
MFGE8	62.0	30.140 to 126.459	77.4	66.7	29.949 to 89.395	36.6	0.553
MGP	72.3	23.484 to 127.995	81.7	79.2	34.839 to 99.635	44.5	0.755
OGN	1.6	0.704 to 3.954	67.9	1.0	0.152 to 2.379	1.7	0.180
PCOLCE	33.6	24.591 to 63.403	78.9	50.1	25.181 to 122.655	61.6	0.755
PLG	26.1	16.152 to 30.258	10.6	21.6	17.681 to 28.366	9.3	0.003
PRG4	1.6	1.217 to 3.365	6.3	1.0	0.447 to 3.273	1.5	0.180
S100A1	9.9	4.346 to 20.051	8.9	6.8	3.074 to 10.283	4.1	0.006
S100A10	7.7	5.317 to 14.731	6.2	7.5	3.330 to 9.826	3.9	0.005

S100A11	12.4	10.769 to 15.641	10.1	12.2	7.684 to 15.241	6.4	0.317
S100A13	11.6	9.969 to 14.431	2.6	14.1	8.504 to 19.672	6.1	0.005
S100A4	6.2	4.369 to 9.654	48.0	7.8	4.704 to 12.724	5.6	0.763
S100A6	154.9	117.629 to 190.838	92.9	199.8	113.263 to 340.135	138.3	0.239
SERPINA1	4.1	2.017 to 7.154	12.5	3.8	2.238 to 5.644	3.7	0.206
SERPINA3	24.5	1.159 to 38.832	24.0	29.2	12.586 to 48.526	17.1	0.011
SERPINA4	4.8	2.352 to 7.620	3.7	4.3	2.817 to 6.200	3.4	0.705
SERPINA5	20.2	14.069 to 52.409	22.0	21.1	10.780 to 31.365	11.7	0.245
SERPINF1	22.6	13.604 to 55.641	42.3	22.3	12.149 to 32.348	12.5	0.246
SERPING1	21.7	15.528 to 53.949	22.0	22.7	13.363 to 37.768	13.1	0.408
SERPINH1	77.3	50.278 to 112.844	39.3	110.9	55.686 to 151.427	55.9	0.167
SMOC1	31.5	12.838 to 45.927	25.7	56.6	37.032 to 115.747	58.8	0.082
SMOC2	37.8	8.944 to 60.586	25.4	85.9	43.725 to 131.630	65.1	0.032
SRPX2	34.3	8.878 to 64.960	28.1	24.7	5.881 to 29.614	12.5	0.089
THBS1	25.5	9.054 to 66.608	29.8	25.4	7.594 to 31.889	13.3	0.227
THBS2	50.8	12.474 to 103.505	46.3	37.6	17.042 to 49.996	19.7	0.168
TIMP1	89.4	59.288 to 138.750	79.4	114.5	55.236 to 165.966	62.8	0.723
VCAN	2.5	1.600 to 4.410	45.6	1.9	0.595 to 3.508	1.9	0.180
VTN	190.6	94.396 to 322.248	122.6	178.2	105.156 to 343.316	132.1	0.844
