# Supplementary material for: Proteomic Analysis of the Meniscus Cartilage in Osteoarthritis
Source: Int J Mol Sci. 2021 Jul 30;22(15):8181. doi: 10.3390/ijms22158181 (PMC8348647; doi:10.3390/ijms22158181)
Supplement: Supplementary file 1 [file ijms-22-08181-s001.zip › Supplementary Table S5.pdf]

Supplementary Table S5. Current researches in pairwise comparison of the L/M OA knee

PMID	Year	Target	Samples	OA (L/M)	Control (L/M)
31249374[1]	2019	gene	multipotential stromal cells from lateral/ medial condyles	17/17	Not performed
33178674[2]	2020	gene	synovial fluid multipotent stromal cells from lateral/ medial knee of OA and following knee joint distraction	21/21	Not performed
32407894[3]	2020	protein	lateral/medial menisci	7/ 9	7/ 9
Current study		protein	lateral/medial menisci	12/12	Not performed

1. Sanjurjo-Rodriguez, C.; Baboolal, T. G.; Burska, A. N.; Ponchel, F.; El-Jawhari, J. J.; Pandit, H.; McGonagle, D.; Jones, E., Gene expression and functional comparison between multipotential stromal cells from lateral and medial condyles of knee osteoarthritis patients. *Scientific reports* **2019,** 9, (1), 9321.
2. Sanjurjo-Rodriguez, C.; Altaie, A.; Mastbergen, S.; Baboolal, T.; Welting, T.; Lafeber, F.; Pandit, H.; McGonagle, D.; Jones, E., Gene Expression Signatures of Synovial Fluid Multipotent Stromal Cells in Advanced Knee Osteoarthritis and Following Knee Joint Distraction. *Frontiers in bioengineering and biotechnology* **2020,** 8, 579751.
3. Folkesson, E.; Turkiewicz, A.; Ali, N.; Ryden, M.; Hughes, H. V.; Tjornstrand, J.; Onnerfjord, P.; Englund, M., Proteomic comparison of osteoarthritic and reference human menisci using data-independent acquisition mass spectrometry. *Osteoarthritis and cartilage* **2020,** 28, (8), 1092-1101.
