# Supplementary figures and images for: Proteomic Analysis of the Meniscus Cartilage in Osteoarthritis
Source: Int J Mol Sci. 2021 Jul 30;22(15):8181. doi: 10.3390/ijms22158181 (PMC8348647; doi:10.3390/ijms22158181)

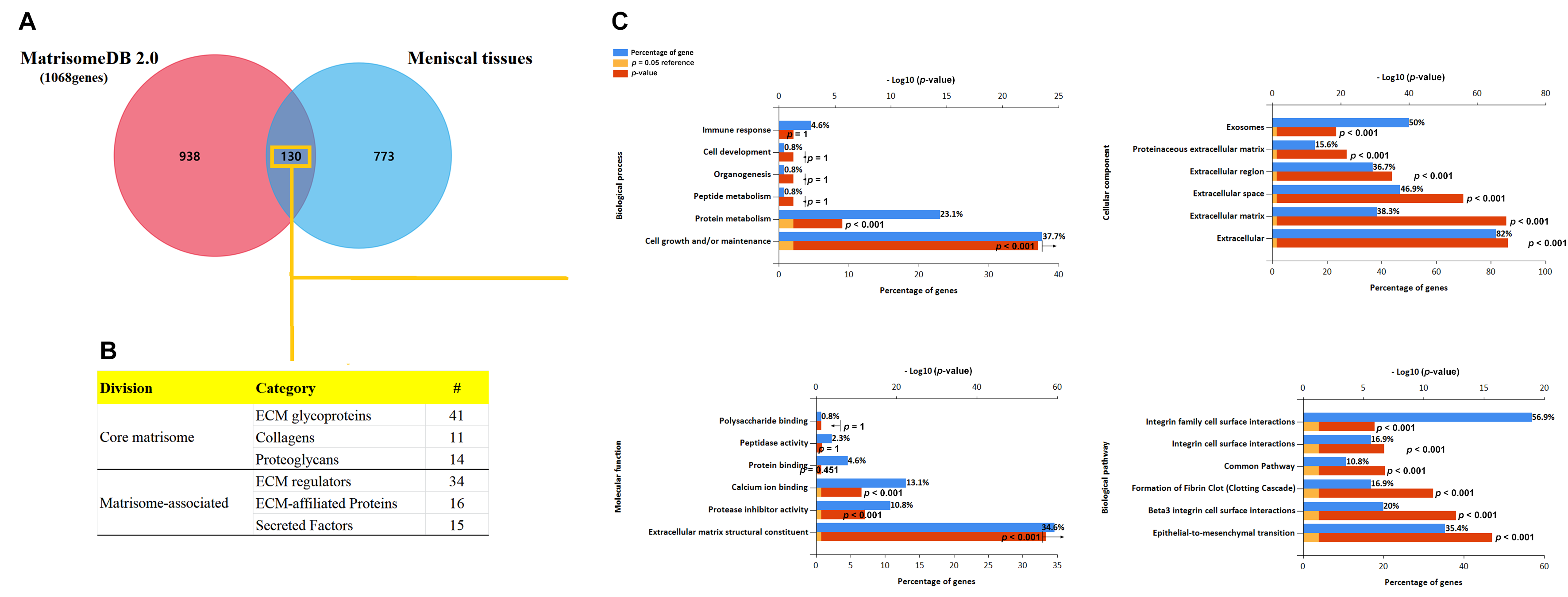

Supplement: Supplementary file 1 [file ijms-22-08181-s001.zip › Fig S1.tif]
